# Neutralization of Acidic Intracellular Vesicles by Niclosamide Inhibits Multiple Steps of the Dengue Virus Life Cycle *In Vitro*

**DOI:** 10.1038/s41598-019-45095-1

**Published:** 2019-06-18

**Authors:** Eunhye Jung, Sangwoo Nam, Hyeryeon Oh, Sangmi Jun, Hyun-Joo Ro, Baek Kim, Meehyein Kim, Yun Young Go

**Affiliations:** 1 Virus Research Group, Korea Research Institute of Chemical Technology, Daejeon, Republic of Korea; 2 Convergent Research Center for Emerging Virus Infection, Korea Research Institute of Chemical Technology, Daejeon, Republic of Korea; 3 Drug and Disease Target Group, Korea Basic Science Institute, Cheongju, Republic of Korea; 4 Center for Drug Discovery, Department of Pediatrics, School of Medicine, Emory University, Atlanta, GA 30322 USA; 5 Department of Pharmacy, College of Pharmacy, Kyung Hee University, Seoul, Republic of Korea; 6 Department of Medicinal Chemistry and Pharmacology, University of Science and Technology, Daejeon, Republic of Korea; 7 Bio-Analytical Science, University of Science and Technology, Daejeon, Republic of Korea

**Keywords:** Antiviral agents, Virology

## Abstract

Dengue fever is one of the most important mosquito-borne viral infections in large parts of tropical and subtropical countries and is a significant public health concern and socioeconomic burden. There is an urgent need to develop antivirals that can effectively reduce dengue virus (DENV) replication and decrease viral load. Niclosamide, an antiparasitic drug approved for human use, has been recently identified as an effective antiviral agent against a number of pH-dependent viruses, including flaviviruses. Here, we reveal that neutralization of low-pH intracellular compartments by niclosamide affects multiple steps of the DENV infectious cycle. Specifically, niclosamide-induced endosomal neutralization not only prevents viral RNA replication but also affects the maturation of DENV particles, rendering them non-infectious. We found that niclosamide-induced endosomal neutralization prevented E glycoprotein conformational changes on the virion surface of flaviviruses, resulting in the release of non-infectious immature virus particles with uncleaved pr peptide from host cells. Collectively, our findings support the potential application of niclosamide as an antiviral agent against flavivirus infection and highlight a previously uncharacterized mechanism of action of the drug.

## Introduction

Dengue virus (DENV) represents a major mosquito-borne pathogen responsible for significant public health and socioeconomic burden in large regions of tropical and subtropical countries^1,2^. There are four distinct serotypes, DENV-1 to DENV-4, that are transmitted mainly by mosquitoes of *Aedes* species, which continuously spread to new geographical areas around the world^3^. The World Health Organization estimates a prevalence of 50–100 million cases of DENV infection per year; however, a recent global estimate study suggested that 390 million DENV infections occur annually, of which 96 million cases have clear symptoms^2^. DENV infection causes a wide range of clinical symptoms, from acute febrile illness (dengue fever) to life-threatening haemorrhagic fever/dengue shock syndrome^1^. To date, clinically approved therapeutic options for treating DENV-infected patients are still lacking.

DENV is an enveloped, single-stranded, positive-sense RNA virus that belongs to *Flavivirus* genus in the family *Flaviviridae*^4^. The genus *Flavivirus* comprises many important emerging arboviruses including Japanese encephalitis virus, West Nile virus and Zika virus (ZIKV). Recently, ZIKV infection has emerged as a global public health concern due to its association with newborn microcephaly^5,6^ and neurological sequelae such as Guillain-Barré syndrome, meningoencephalitis, and myelitis in infected adults^6–10^. The flavivirus genome is approximately 11 kb in length and encodes a polyprotein that is processed into three structural (capsid [C], premembrane [prM], and envelope [E]) and seven non-structural proteins (NS1, NS2A, NS2B, NS3, NS4A, NS4B, and NS5) by cellular and viral proteases^11,12^. Flavivirus infection is initiated by attachment of the virus to a cellular receptor on the plasma membrane followed by receptor-mediated endocytosis and transportation of viral particles to endosomes^13,14^. Viral membrane fusion with the endosomal membrane is triggered upon exposure of the virus to the low-pH environment of endosomes, through which the viral genome is released into the cytoplasm^15–19^. Following RNA replication and protein translation, immature virions containing prM proteins are assembled within the endoplasmic reticulum (ER) and mature through passaging the acidic environment of the trans-Golgi network (TGN), wherein E proteins undergo conformational changes and the pr peptides are cleaved by furin endoproteases, after which progeny virions are released from the host cell^20–24^.

It is well established that neutralization of the acidic TGN environment prevents furin cleavage, resulting in immature particles containing uncleaved prM proteins^25–27^. These immature particles are non-infectious since the uncleaved prM peptides block the low-pH-induced conformational changes of the viral E proteins essential for binding to the cell surface as well as membrane fusion of the virus during entry^23,26,28,29^. Thus, several studies have shown that lysosomotropic agents, such as chloroquine, exert modest antiviral effects against pH-dependent viruses, including flaviviruses, by interfering with endosomal fusion and furin-dependent maturation *in vivo* and *in vitro*^23,30–32^. Recently, niclosamide, a U.S. Food and Drug Administration (FDA)-approved antiparasitic drug used in humans^33–35^, has been identified as an effective antiviral agent against a number of pH-dependent viruses, such as human rhinoviruses and influenza virus^36^, severe acute respiratory syndrome-coronavirus^37^, Chikungunya virus^38^, and flaviviruses^39–41^. These studies suggested that the broad antiviral activity of niclosamide is associated with neutralization of endo-lysosomal pH that interferes with pH-dependent membrane fusion which is a critical step for virus entry^36^. In a recent study, Kao *et al*.^41^, determined the inhibitory role of endosomal deacidification in DENV viral genome replication and uncoating but not in later steps of the viral life cycle. Therefore, the possible effect of niclosamide-induced neutralization of endosomal compartments on later stages of the DENV infectious cycle remains to be elucidated.

In this study, we investigated and confirmed that the neutralization of low-pH intracellular compartments by niclosamide affects multiple steps of the DENV infectious cycle. Our data indicate that niclosamide-induced endosomal neutralization prevents viral genome release and replication as well as maturation of DENV particles rendering it non-infectious. Specifically, we found that niclosamide-induced endosomal neutralization blocks conformational changes of E glycoproteins on the virion surface of both DENV and ZIKV, resulting in the release of immature virus particles with uncleaved pr peptide and preventing them from infecting new host cells. Collectively, our findings support the potential application of niclosamide as an antiviral agent against flavivirus infection and highlight a previously uncharacterized mechanism of action of the drug.

## Results

### Niclosamide inhibits infection by all four serotypes of DENV in Huh-7 cells

**Figure 1:**
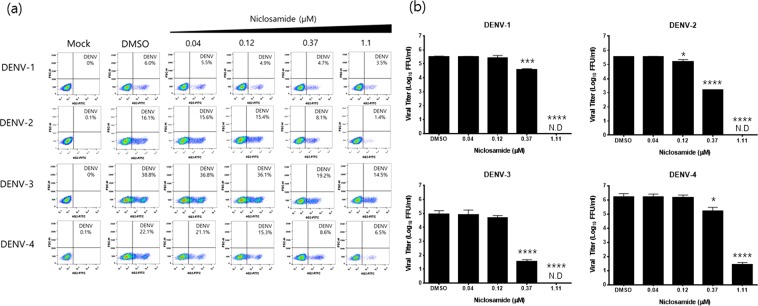
Antiviral activity of niclosamide against DENV-1-4 in Huh-7 cells. (**a**) Representative dot plot analysis (FSC x 4G2-AF488) of Huh-7 cells infected with DENV-1, DENV-2, DENV-3, or DENV-4 in the presence of the indicated concentrations of niclosamide. The DENV-positive cell population is shown in the lower right quadrant. **(b)** Viral titres from cell culture supernatants were determined with a focus-forming assay. The data represent the means (±SD) of at least two independent experiments performed in duplicate. N.D., not detected. *p < 0.05, ***p < 0.001 and ****p < 0.0001 compared to DMSO control.

In this study, the anti-dengue activity of niclosamide was evaluated using four DENV serotypes. After 24 h post-infection (p.i), viral titres from the supernatants and the number of infected cells were measured by fluorescence-activated cell sorter (FACS) analysis and focus-forming assay, respectively. The proportion of cells positive for DENV antigen decreased in a dose-dependent manner in cells treated with niclosamide compared to that in DMSO-treated controls as determined by FACS analysis (Fig. 1a). Specifically, the percentage of DENV-positive cells was significantly reduced when infected cells were treated with niclosamide at a concentration of 0.37 μM or higher for all four serotypes of DENV (Fig. 1a). The EC_50_ values of niclosamide against DENV-1, DENV-2, DENV-3, and DENV-4 were approximately 1.45 μM, 0.38 μM, 0.37 μM and 0.25 μM, respectively. To rule out the possibility that virus infected cells were more sensitive to niclosamide treatment resulting in synergistic cytotoxicity compared to mock-infected cells, the percentages of live and dead cells from mock- and DENV-2 infected cultures with increasing concentrations of niclosamide were determined by FACS analysis. The data showed no difference in cell viability between mock- and DENV-infected cells treated with increasing concentrations of niclosamide (Supplementary Fig. S1). Next, the 50% cytotoxicity concentration (CC_50_) value of niclosamide was determined by measuring cell viability using the MTT assay. The estimated CC_50_ value of niclosamide was >10 μM; however, minor cytotoxic effects at all sub-lethal doses were observed in Huh-7 cells (Supplementary Fig. S2). Similarly, niclosamide inhibited the production of infectious DENV particles of all four serotypes in a dose-dependent manner as quantified by the focus-forming assay. Significantly, no infectious DENV particles were detected when infected Huh-7 cells were treated with niclosamide at a concentration of 1 μM or higher (Fig. 1b). These results together confirm that niclosamide effectively inhibits DENV infection independent of the virus serotype within a non-cytotoxic range in Huh-7 cells.

### Niclosamide inhibits both the early and late stages of the DENV infectious life cycle

**Figure 2:**
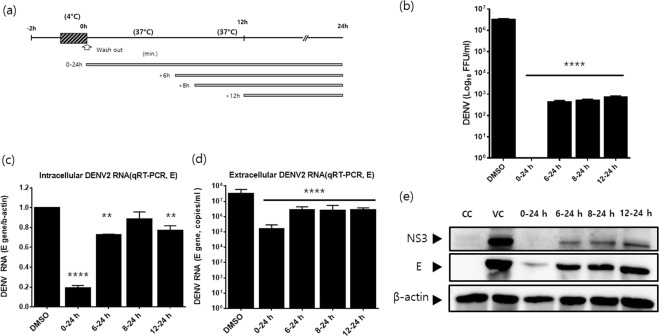
Niclosamide affects both the early and late stages of dengue virus replication. **(a)** Schematic representation of the time-of-addition experiment. Niclosamide was added to DENV-2-infected cells at the indicated time points and remained present until sample collection. DMSO was used as a control. **(b)** Infectious virus titres in the supernatants were determined by focus-forming assay. **(c** and **d)** Parallel infections were collected for quantification of intracellular and extracellular DENV-2 RNAs by RT-qPCR**. (c)** Intracellular viral RNA levels (*E* gene) were normalized to *β-actin* mRNA. **(d)** Extracellular viral RNA levels were expressed as mRNA copy numbers per ml (copies/ml). **(e)** DENV-2 NS3 and E protein levels in lysates of infected cells were determined by Western blot analysis. Different probing is divided by white space. Uncropped images are presented in the supplemental material. CC; cell control. VC; virus control. The data represent the means (±SD) of at least two independent experiments performed in duplicate. **p < 0.01 and ****p < 0.0001 compared to DMSO control.

After demonstrating that niclosamide has an antiviral effect against DENV independent of its serotype, the DENV-2 NGC strain was employed in all subsequent experiments. We performed a time-of-addition experiment to investigate the stage of the DENV life cycle during which niclosamide exerts its antiviral activity. Huh-7 cells were infected with DENV-2 and subsequently treated with 1 μM niclosamide starting at 0, 6, 8 and 12 h p.i. until the medium and cell lysates were harvested at 24 h p.i. (Fig. 2a). Analysis of the infectious progeny released in the supernatant revealed that niclosamide not only affected an early stage of the viral life cycle but also reduced progeny titres when added at later stages. Specifically, there was a complete inhibition of infectious virus production when niclosamide was added at 0 h p.i., while a 3-log reduction was observed when niclosamide treatment was initiated at 6, 8 or as late as 12 h p.i. (Fig. 2b). Similarly, niclosamide treatment was maximally effective (~80%) in reducing intracellular viral RNA when added at 0 h p.i., while the inhibitory effect markedly decreased when the drug was added at later time points (6, 8 and 12 h p.i.), suggesting that niclosamide interferes with viral RNA replication (Fig. 2c). Likewise, there was a marked reduction of viral genome copies released in the supernatant from samples that received niclosamide treatment at 0 h p.i. (~ 2-log reduction), while the effect diminished when the drug was added at later time points such as 6, 8 or as late as 12 h p.i. (~1-log reduction, Fig. 2d). Thus, the data together indicated that niclosamide possibly inhibits viral RNA replication when it is added at early step of viral life cycle but it also affects a late stage of virion biogenesis, such as maturation, since a strong antiviral effect was consistently observed in infected cells with niclosamide treatment starting as late as 12 h p.i. (Fig. 2b–d). In addition, viral NS3 and E protein levels were significantly reduced upon niclosamide treatment at early post-infection time points, whereas the antiviral effect diminished when the drug was added after 6 h p.i. (Fig. 2e). Taken together, these data suggest that niclosamide potently inhibits the early stages of the DENV life cycle, which impacts viral RNA accumulation and protein expression, as well as the production of infectious viruses. In addition, the data revealed that niclosamide affects not only an early stage but also a late stage of the DENV life cycle independent of the effects on intracellular viral RNA accumulation and protein synthesis.

### Neutralization of endosomal pH by niclosamide inhibits DENV RNA genome replication and viral polyprotein processing

**Figure 3:**
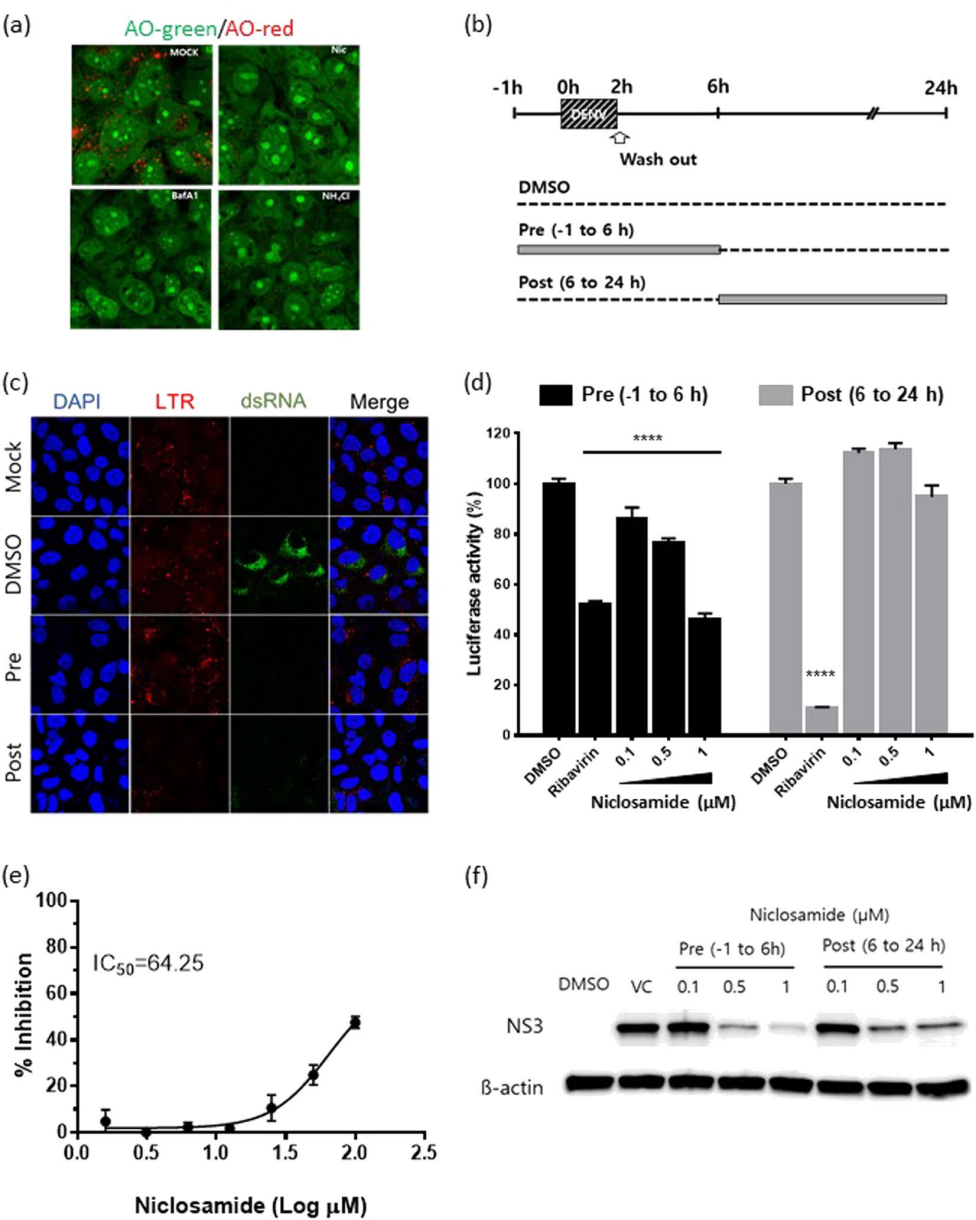
Antiviral activity of niclosamide is associated with neutralization of endosomal pH. (**a**) Representative ratiometric live cell image of AO staining showing acidic compartments (*red*) in cells treated with niclosamide, BafA1 or NH_4_Cl. **(b)** Schematic representation of the time-of-addition experiment. **(c)** Representative immunofluorescence staining images of acidic compartments with LysoTracker (LTR, *red*) probe and viral dsRNA (*green*) in the presence of niclosamide at different time points. **(d)** Luciferase activity in BHK-D2-Rluc replicon cells treated with different concentrations of niclosamide at the indicated times. The data represent the means (±SD) of at least two independent experiments performed in duplicate. **(e)** Dose-response inhibition of *in vitro* DENV-2 NS2B-NS3 proteolytic activity by niclosamide. **(f)** Western blot analysis of DENV NS3 protein levels in lysates of infected cells that were treated with different concentrations of niclosamide at the indicated times. Immunoblot detection of β-actin is shown as a loading control. DMSO was used as the solvent control. Different probing is divided by white space. Uncropped images are presented in the supplemental material. VC; virus control. ****p < 0.0001 compared to DMSO control.

Niclosamide is a proton carrier and blocks endosomal acidification^36^. We tested whether niclosamide neutralizes the low-pH compartments in Huh-7 cells by using acridine orange (AO), which is a fluorescent pH-sensitive dye. As shown in Fig. 3a, the low pH of endosomes (red) present in mock-treated Huh-7 cells was completely absent in cells treated with niclosamide (green), suggesting that it effectively blocked endosomal acidification in Huh-7 cells. Bafilomycin A1 (BafA1) and ammonium chloride (NH_4_Cl) were used as controls. To further confirm the correlation of the antiviral effect with the neutralization of endosomal pH, lysotracker (a marker for acidic compartments) analysis was performed together with immunofluorescence staining for the detection of double-stranded RNA (dsRNA) in DENV-infected cells treated with niclosamide at early (pre, −1 h to 6 h p.i.) or late stages (post, 6 h p.i. to 24 h p.i.) (Fig. 3b). The results showed that viral dsRNA expression, an intermediate product in replication, was significantly inhibited in infected cells treated with niclosamide at early and late stages compared to that in DMSO-treated controls, indicating that viral RNA replication is severely affected (Fig. 3c). Notably, there was no difference in the levels of endosomal acidification in cells treated with niclosamide during the early stage and subsequently removed from the culture, whereas the endosomal pH was effectively neutralized in cells that received the treatment at the late stage and the treatment remained until lysotracker staining, indicating the reversible effect of the drug on the inhibition of endosomal acidification (Fig. 3c). To further corroborate that suppression of viral genome replication is specifically due to niclosamide-induced pH neutralization, DENV replicon reporter BHK21 cells encoding the renilla luciferase gene were treated with niclosamide at the time points indicated in Fig. 3b. Ribavirin, a broad-spectrum antiviral compound known to impede RNA virus replication, was used as a positive control. Cell viability of replicon cells in the presence of niclosamide and ribavirin treatment was determined by the MTT assay (Supplementary Fig. S3). As shown in Fig. 3d, treatment of DENV replicon reporter cells with 1 μM niclosamide at early time points (pre, −1 h to 6 h) resulted in a significant reduction in luciferase activity, comparable to that of ribavirin, whereas the inhibitory effect was almost restored to the level of DMSO-treated cells when the cells were treated with niclosamide after 6 h of culture (post, 6 h p.i. to 24 h p.i). In contrast, the reduction in luciferase activity reached a maximum when ribavirin was added at 6 h p.i. and remained in the culture until harvest. Thus, the replicon-based assay suggested that niclosamide inhibits the steps involved in viral RNA replication independent of its effect on entry, membrane fusion and genome release. Next, we evaluated the impact of niclosamide on the proteolytic activity of DENV NS2B-NS3, which plays an essential role in viral polyprotein processing required for the initiation of viral replication. The proteolytic activity of DENV NS2B-NS3 protease, using the fluorogenic substrate, was evaluated in the presence of various concentrations of niclosamide. As a result, the half-maximal inhibitory concentration (IC_50_) for niclosamide was determined to be 64.2 μM, showing only a modest inhibition of NS3 protease activity (Fig. 3e). Lastly, the expression level of DENV non-structural (NS3) protein after niclosamide treatment at early (pre, −1 h to 6 h p.i.) or late stages (post, 6 h p.i. to 24 h p.i.) was examined by Western blot analysis (Fig. 3f). Consistent with the immunofluorescence assay results, the expression of NS3 protein was significantly reduced in cells treated at early and late stages, although the inhibition was more pronounced in cells treated at the early stage of virus replication than in those treated after 6 h p.i. (Fig. 3f). Overall, the data indicate that the antiviral activity of niclosamide during the early stage of the DENV life cycle correlates with the neutralization profile of the low-pH compartments, suggesting that blocking endosomal acidification results in the inhibition of viral genome replication and polyprotein processing, which further impedes viral protein expression and virus production.

### Neutralization of low-pH compartments by niclosamide also impairs the DENV maturation process

**Figure 4:**
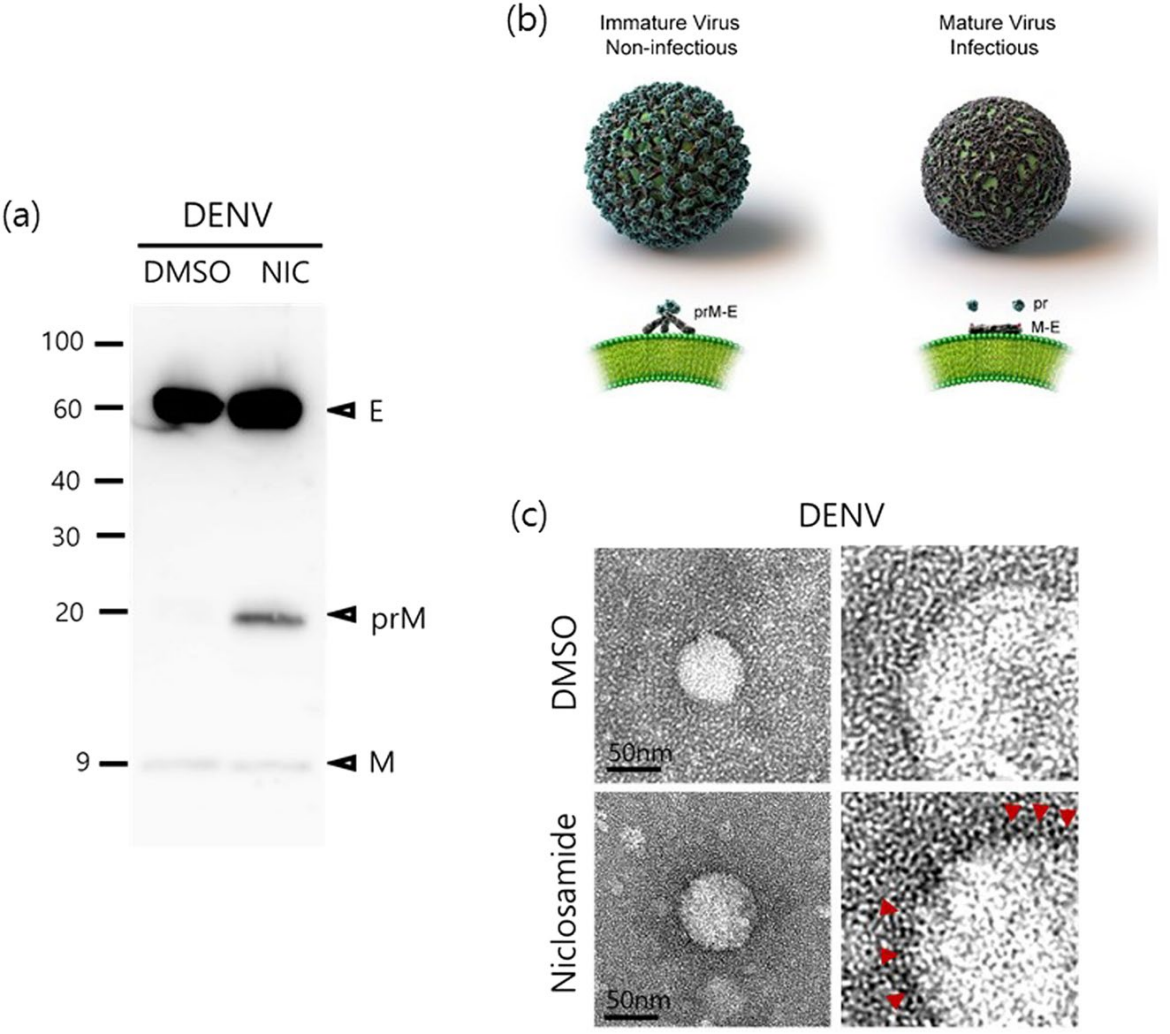
Niclosamide-induced endosomal pH neutralization impairs the maturation of DENV. (**a)** Western blot analysis of DENV-2 prM and E proteins purified from supernatants treated with niclosamide or DMSO. Uncropped images are presented in the supplemental material. **(b)** Structural representation of dengue virion and conformation of E proteins. **(c)** Representative TEM images of DENV particles purified from culture supernatants treated with niclosamide or DMSO.

To further corroborate the effect of niclosamide-induced neutralization of low-pH compartments on virion biogenesis, particularly maturation, which is a pH-dependent process, we analysed the composition of prM and E proteins in the virus particles produced by infected cells treated with niclosamide. Briefly, DENV-2-infected Huh-7 cells were treated with 1 μM niclosamide at 6 h p.i. and supernatants were collected at 30 h p.i. Subsequently, tissue culture medium obtained from niclosamide-treated and mock-treated cells was pelleted by ultracentrifugation. Samples were adjusted to equal viral genomic copy numbers, separated by SDS-PAGE, and viral proteins were detected by Western blot using monoclonal antibodies against E and prM proteins. The data showed that virus particles obtained from niclosamide-treated and untreated (DMSO) samples had comparable levels of DENV E protein (Fig. 4a). In contrast, a clear prM protein band (approximately 20 kDa) was detected in DENV released from niclosamide-treated cells, in which the pr peptide was not cleaved during maturation, resulting in the release of immature and non-infectious virus particles in the medium. In contrast, the prM protein band was barely detected from virions released from DMSO-treated cells, indicating that they were mature and fully infectious (Fig. 4a). Next, the morphology of DENV particles released from DMSO- and niclosamide-treated cells was examined by transmission electron microscopy (TEM). As shown in Fig. 4, DENV particles derived from DMSO-treated cells had relatively smooth and spike-less outer surfaces, which are characteristics of mature particles (Fig. 4b,c [upper panels]) compared to those obtained from niclosamide-treated cells, which had the expected spiky appearance of immature particles (Fig. 4b,c [lower panels]). Similar results were observed in human monocytic U937-dendritic cell-specific ICAM-grabbing non-integrin (U937-DC-SIGN) cells suggesting that the antiviral effect of niclosamide is not limited to Huh-7 cells (Supplementary Fig. S4). Taken together, these results indicate that inhibition of endosomal acidification by niclosamide interferes with the pH-dependent DENV maturation process by preventing cleavage of the pr peptide from the M protein on surface of the virus.

### The ZIKV particle maturation process is affected

**Figure 5:**
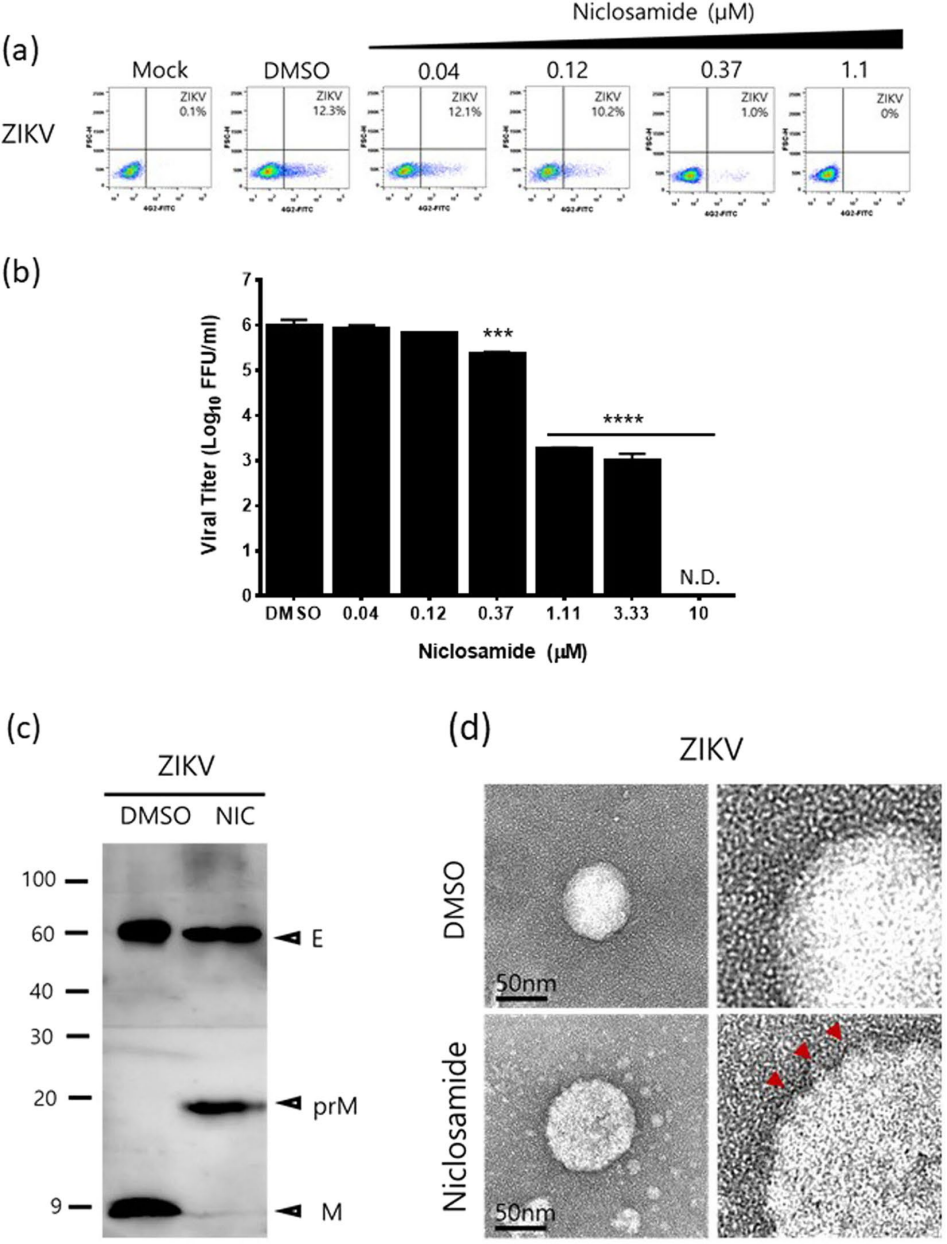
Niclosamide-induced endosomal pH neutralization impairs the maturation of ZIKV. **(a)** Representative dot plot analysis (FSC x 4G2-AF488) of Huh-7 cells infected with ZIKV in the presence of the indicated concentrations of niclosamide. The ZIKV-positive cell population is shown in the lower right quadrant. **(b)** Virus titres from cell culture supernatants were determined with a plaque assay. The data represent the means (±SD) of at least two independent experiments performed in duplicate. N.D., not detected. **(c)** Western blot analysis of prM and E proteins of ZIKV purified from culture supernatants treated with niclosamide or DMSO. Uncropped images are presented in the supplemental material. **(d)** Representative TEM images of ZIKV particles purified from culture supernatants treated with niclosamide or DMSO. ***p < 0.001 and ****p < 0.0001 compared to DMSO control.

We hypothesized that niclosamide-induced neutralization of low-pH compartments also impacts ZIKV, a member of the *Flavivirus* genus, virion biogenesis. We first corroborated that niclosamide reduced the number of ZIKV-positive cells and infectious viral production in a dose-dependent manner in Huh-7 cells with an EC_50_ of 0.2 μM as evaluated by FACS analysis and focus-forming assay, respectively (Fig. 5a,b). Next, the composition of prM and E proteins in ZIKV virions released in the medium of niclosamide-treated cells was examined to confirm whether niclosamide impacts the maturation process. As shown in Fig. 5c, ZIKV particles released in the medium of DMSO-treated cells were completely processed with no detectable prM protein band, while a prominent M protein band at approximately 9 kDa was observed, indicating that the virions are mature and fully infectious. In contrast, ZIKV particles released in the medium of niclosamide-treated cells had a prominent prM protein band, and almost no M protein was detected, indicating that cleavage of the pr peptide during the maturation process had been hampered (Fig. 5c). Consistent with these results, the electron microscopy images also demonstrated that virions from DMSO-treated cells had a smoother surface (Fig. 5d, upper panels), while those obtained from niclosamide-treated cells had a spiky outer surface (Fig. 5d, lower panels), indicating that conformational surface protein changes and pr peptide cleavage were blocked. Taken together, our data suggest that niclosamide-induced neutralization of low-pH compartments interferes with the DENV and ZIKV maturation process, resulting in the release of prM-containing immature and non-infectious viral particles into the extracellular environment.

## Discussion

Niclosamide is a well-established drug that has been safely used for antihelmintic therapy against tapeworm infection for approximately 50 years^33^. Its antiparasitic activity is attributed to its ability to inhibit mitochondrial oxidative phosphorylation and anaerobic ATP production, which affect the pH homeostasis of parasites^42,43^. Recently, niclosamide has also been identified as an effective antiviral agent against a number of pH-dependent viruses. Its inhibitory effect has been attributed to the neutralization of endo-lysosomal pH, which interferes with pH-dependent membrane fusion that is critical for virus entry^36^. In this study, we found that neutralization of low-pH intracellular compartments by niclosamide not only inhibited the early stage of the DENV viral life cycle, such as viral RNA replication, independent of the entry step but also the late stage, specifically, the maturation of virus particles into infectious virions.

Similar to previous reports, our data showed that niclosamide neutralized the low-pH intracellular compartments in a reversible manner and suggested that its antiviral effect correlates with neutralization of these acidic environments, which are critical for DENV replication^36,41^. Here, we confirmed that niclosamide effectively inhibited intracellular viral RNA synthesis, protein expression and production of infectious DENV particles when the drug was added at an early time point during infection. The immunofluorescence assay coupled with staining of intracellular acidic compartments in live DENV-infected cells showed that niclosamide-induced pH neutralization during the first 6 h of infection was sufficient to completely block viral dsRNA replication and its subsequent steps, such as viral protein expression and production of infectious viral particles. Indeed, niclosamide treatment during the first 6 h of infection reduced DENV replication in BHK-21 cells harbouring dengue replicons to a level comparable to that of ribavirin, suggesting that the drug affects viral RNA replication and/or translation independent of its effect on entry, membrane fusion and genome release. Furthermore, we provide evidence that niclosamide impairs the proteolytic activity of DENV NS2B-NS3, which is essential for the initiation of viral replication and may explain its effects on DENV genome replication and/or translation, strengthening the findings of Li *et al*.^44^. However, these findings are somewhat contradictory to the results reported by Kao *et al*.^41^, which, using a replicon-based assay, indicated that niclosamide had no effect on DENV genome replication. We speculate that the variance in the efficacy of niclosamide against viral genome replication using replicon-based cells could be due to a difference in the times of drug-addition used in each study.

In this study, we identified a new potentially interesting mode of action of niclosamide, which impacts a late stage of the virus life cycle in Huh-7 cells. Our time-of-addition data demonstrated that the quantity of infectious virus particles released into the media was significantly reduced even when niclosamide was added to the cells several hours after infection, whereas intracellular and extracellular viral RNA levels were not affected to the same extent as the production of infectious virus particles. These data led us to the hypothesis that niclosamide-induced intracellular pH neutralization affects the maturation of DENV particles. It has been extensively described that the low-pH-induced conformational rearrangement of the prM and E proteins is critical for DENV maturation and infectivity^23,26–29,45–50^. Indeed, our data showed that DENV, as well as ZIKV particles obtained from niclosamide-treated cells, contained high levels of uncleaved prM proteins compared to the untreated virions, suggesting that most of the virus particles were immature and non-infectious. Similarly, transmission electron microscopy images showed that the DENV and ZIKV particles secreted from niclosamide-treated cells were substantially larger than untreated control virions and had irregularities on the surface, indicating that the viral particles were immature and contained uncleaved prM on the surface. Taken together, we believe that we have provided the first evidence that neutralization of the low-pH intracellular compartments by niclosamide prevents conformational changes to E glycoproteins on the virion surface during the flavivirus maturation process, which results in the release of immature non-infectious virus particles from host cells.

**Figure 6:**
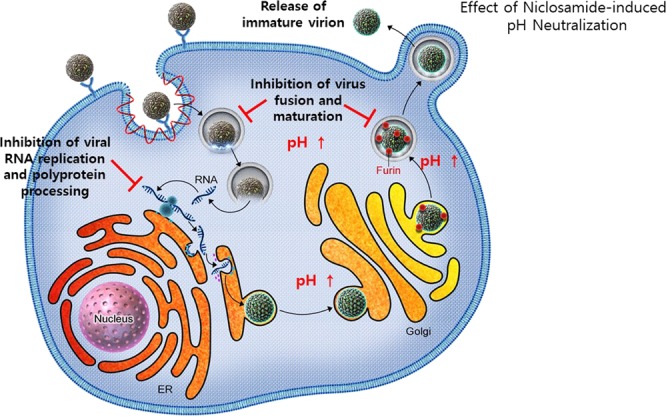
Schematic representation of flavivirus life cycle and proposed antiviral mechanism of niclosamide. Niclosamide-induced neutralization of the low-pH intracellular organelles inhibits multiple steps of DENV life cycle, including viral RNA replication and polyprotein processing, virus fusion and maturation of progeny virions.

In summary, we confirmed that the antiviral effect of niclosamide against flaviviruses is mostly associated with neutralization of the low-pH intracellular organelles. As a consequence, multiple pH-dependent steps of the flavivirus life cycle, including viral and host membrane fusion and uncoating, viral RNA replication (by inhibition of viral polyprotein processing), and the maturation process of the progeny virions, are impaired, highlighting the complexity of the antiviral efficacy of niclosamide (Fig. 6). Collectively, the data presented in this study provide further evidence to support the repurposing of niclosamide as a potential therapeutic option against pH-dependent RNA viruses, particularly flaviviruses.

## Materials and Methods

### Cells and viruses

C6/36 mosquito cells (ATCC^®^ CRL-1660^TM^) and Vero76 (ATCC^®^ CRL-1587^TM^) cells were maintained in Minimum Essential Medium (MEM; Invitrogen, Carlsbad, CA, USA) supplemented with 10% foetal bovine serum (FBS; HyClone, Salt Lake City, UT, USA) at 28 °C and 37 °C, respectively. Human hepatoma-7 (Huh-7) cells were purchased from Japan Cell Research Bank (National Institutes of Biomedical Innovation, Health and Nutrition, Japan) and maintained in high-glucose Dulbecco’s Modified Eagle’s Medium (DMEM; HyClone) supplemented with 10% FBS at 37 °C. The BHK-21 cells harbouring luciferase-expressing DENV replicons (BHK-D2-Rluc) were maintained in DMEM containing 10% FBS and 5 μg/ml puromycin (InvivoGen, Pak Shek Kok, Hong Kong). DENV-1 (DenKor-02) was obtained from the Korea National Institute of Health (Osong, Republic of Korea)^51,52^. DENV-2 (New Guinea C strain) was purchased from the National Collection of Pathogenic Viruses, Culture Collections of Public Health England (Salisbury, Great Britain). DENV-3 (DENV-3/KBPV-VR-30) and DENV-4 (DENV-4/KBPV-VR-31) were purchased from the Korea Bank for Pathogenic Viruses (KBPV, Seoul, Republic of Korea)^53^. All DENVs were propagated in C6/36 cells, and tissue culture fluid (TCF) supernatants were harvested at 6–7 days post-infection. TCF was clarified by centrifugation, and 1-ml aliquots were stored at −80 °C until further use. Viral titres were quantified by focus-forming assay on Vero76 cells as described previously^54^. Zika virus, an MR766 strain (ATCC^®^ VR-1838^TM^) purchased from ATCC, was amplified, and viral titres were measured by plaque assay on Vero76 cells.

### Antiviral assay

To determine the antiviral activity of niclosamide, Huh-7 cells were infected with DENV-1, DENV-2, DENV-3, DENV-4, or ZIKV at a multiplicity of infection (MOI) of 0.5 and treated with either niclosamide (Sigma-Aldrich, St. Louis, MO, USA) or a solvent control, dimethyl sulfoxide (DMSO; Sigma-Aldrich), at the indicated concentrations. Dose-response curves were obtained with 3-fold serial dilutions of niclosamide ranging from 0.04 μM to 10 μM. At 24 h p.i., live cells and cell culture supernatants were collected and processed for flow activated cell sorter (FACS) analysis and virus titration, respectively.

### Flow cytometry analysis

Cells were centrifuged at 500 × *g* for 5 min and resuspended in 200 μl PBS. Cells were stained with eBioscience Fixable Viability Dye eFluor 780 (Invitrogen) on ice for 30 min and washed twice with PBS. Subsequently, cells were incubated in Cytofix/Cytoperm solution (BD Biosciences, Franklin Lakes, NJ, USA) on ice for 20 min in the dark. Cells were then washed with Perm/Wash Buffer (BD Biosciences) and stained with anti-DENV E (4G2) monoclonal antibody conjugated to AlexaFluor 488 (AF488) for 1 h on ice. Subsequently, cells expressing DENV E were analysed by a FACSAria III flow cytometer (BD Biosciences) using FlowJo^®^ software 10.0.7 (FlowJo, LLC, Ashland, OR, USA). For each sample, at least 20,000 events were collected.

### Time-of-addition assay

To determine the mechanism of action of niclosamide, Huh-7 cells were inoculated with the DENV-2 NGC strain at an MOI of 0.5 at 4 °C for 2 h. Unbound virus was removed by washing with ice-cold phosphate buffered saline (PBS), fresh medium was then added, and plates were shifted to 37 °C to allow synchronous entry and infection. Soon after the temperature shift, 1 µM niclosamide was added at 0, 6, 8 and 12 h and maintained throughout the infection. At 24 h p.i., cell culture supernatants were collected for virus titration and extracellular viral RNA quantification by focus-forming assay and quantitative reverse-transcription polymerase chain reaction (RT-qPCR), whereas cell lysates were harvested and subjected to intracellular viral RNA and viral protein analyses by RT-qPCR and Western blot assays, respectively.

### Quantitative reverse-transcription polymerase chain reaction (RT-qPCR)

For intracellular viral RNA quantification, total cellular RNA was purified from cell lysates using an RNeasy Mini kit (Qiagen, Valencia, CA, USA) according to the manufacturer’s instructions. Viral RNA in the TCF samples was extracted using a QIAamp^®^ Viral RNA Mini kit (Qiagen) according to the manufacturer’s instructions. RT-qPCR was performed using a S*uper*Script III one-step RT-PCR system with Platinum *Taq* polymerase (Invitrogen), primers/probe sets targeting *NS5* or *E* genes and a QuantStudio 6 real-time PCR system (Applied Biosystems, Foster City, CA, USA) as described previously^54^. The relative viral RNA expression levels were calculated by the ΔΔ*C*_*T*_ method, and *β-actin* was used as an endogenous control. Absolute viral RNA genome copy number was calculated based on the *in vitro-*transcribed DENV RNA standard curve and reported as the absolute number of viral RNA genome copies per ml of TCF^54^. Two biological replicates, each with technical duplicates, were used for quantification.

### Western blot analysis

At 24 h p.i., virus-infected cells were washed with PBS and lysed using M-PER buffer (Thermo Fisher Scientific, Waltham, MA, USA) containing 0.5% protease inhibitor cocktail (Pierce, Rockford, IL, USA). The cell lysates were clarified by centrifugation, and the total protein content was determined by the Bradford assay (Bio-Rad, Hercules, CA, USA). Equal amounts of protein were subjected to sodium dodecyl sulfate polyacrylamide gel electrophoresis (SDS-PAGE) and electro-transferred to a PVDF membrane. Viral proteins were detected using primary antibodies specific for DENV NS3, DENV E, DENV prM, ZIKV E, and ZIKV prM followed by a horseradish peroxidase (HRP)-conjugated goat anti-mouse or anti-rabbit secondary antibody. As a loading control, cellular β-actin was detected with an anti-β-actin-specific primary antibody and HRP-conjugated goat anti-mouse secondary antibody. After the addition of a chemiluminescent HRP substrate (SuperSignal West Pico Chemiluminescent Substrate; Pierce), images were obtained using a LAS-4000 Luminescent Image Analyzer (Fujifilm, Tokyo, Japan).

### DENV replicon assay

BHK-21 cells encoding a luciferase-expressing DENV-2 replicon (BHK-D2-Rluc) were used to determine DENV RNA replication efficiency. Briefly, BHK-21 replicon cells were seeded in 96-well plates and incubated with different concentrations of niclosamide at the indicated time points at 37 °C for 24 h. After incubation, antiviral activity was measured using the Renilla Luciferase Assay (Promega, Madison, WI, USA) in a microplate luminometer (Tecan, Männedorf, Switzerland).

### *In vitro* protease activity assay

The DENV-2 NS2B-NS3 protease expression plasmid used in this study was kindly provided by Dr. Rolf Hilgenfeld, University of Lübeck, Lübeck, Germany. The *in vitro* DENV-2 NS2B-NS3 protease activity assay was performed as described elsewhere^55^. The fluorogenic peptide substrate (Boc-Gly-Arg-Arg-AMC) was purchased from Bachem AG (Bubendorf, Switzerland).

### Confocal microscopy

To measure pH changes in cytoplasmic membrane-enclosed vesicles, cells were stained with acridine orange (AO, Thermo Fisher Scientific), as described previously^56^. Briefly, Huh-7 cells (1 × 10^5^ cells per well) were cultured at 37 °C in 35 mm glass-bottom dishes (Greiner Bio-One, Frickenhausen, Germany). On the following day, cells were treated for 1 h with 10 μM niclosamide, 100 nM bafilomycin A1 (a V-ATPase inhibitor), or 50 mM ammonium chloride (an intralysosomal pH-neutralizing agent). Acridine orange was added to the culture medium at a final concentration of 2 μg/ml, and the cells were imaged with a confocal microscope Zeiss LSM700 META (Carl Zeiss, Oberkochen, Germany). The excitation wavelength was 488 nm, and images were collected in two emission windows: 493–560 nm and 590–720 nm.

For labelling and tracking of acidic organelles in niclosamide-treated DENV-infected cells, a deep red-fluorescent dye, LysoTracker DND-99 (LTR, Thermo Fisher Scientific), was used. Briefly, Huh-7 cells were grown on 4-well chamber slide and incubated overnight. The Huh-7 cells were infected with the DENV-2 NGC strain at an MOI of 5 and treated with 1 μM niclosamide at 1 h prior to infection and left in culture until 6 h p.i. (pre −1 to 6 h) or treated at 6 h p.i. and the treatment maintained throughout the course of the infection. After 24 h of incubation, cells were washed and loaded with 0.5 μM Lysotracker DND-99 (Invitrogen) for 30 min at 37 °C. Subsequently, the cells were washed with PBS and fixed with 4% paraformaldehyde in PBS for 30 min at room temperature. The fixed cells were incubated with antibodies against DENV E protein or dsRNA antibodies for 1 h at room temperature. The cells were then incubated with AF488-conjugated goat anti-mouse IgG antibodies for 1 h at room temperature. Nuclei were counterstained by incubation for 5 min with 1 μg/ml DAPI, and the coverslips were mounted in ProLong^TM^ Gold Antifade reagent (Invitrogen). Image analysis was performed using a confocal microscope (Zeiss LSM700 META).

### Electron microscopy

Huh-7 cells were infected with DENV-2 (NGC strain) or ZIKV (MR766 strain) at an MOI of 0.5 for 2 h at 37 °C. At 6 h p.i., 1 μM niclosamide or DMSO was added and infected cultures were further incubated for 24 h. After a total of 30 h of infection, virus particles were pelleted by ultracentrifugation at 4 °C in a Beckman type SW43 rotor at 100,700 × *g* for 4 h. Virion pellets were resuspended in NTE buffer. Five microliters of resuspended DENV-2 and ZIKV were mounted on plasma-cleaned 200 mesh, carbon-coated, copper grids (Electron Microscopy Sciences, Hatfield, PA). Grids were washed once with distilled water and then negatively stained with 2% aqueous uranyl acetate (Electron Microscopy Sciences, Hatfield, PA) for 45 s. The solution was blotted with filter paper, and the sample grids were rinsed briefly with distilled water three times. After drying in air, the grids were examined under a Zeiss LEO912AB Transmission Electron Microscope (Carl Zeiss) at an accelerating voltage of 120 kV and an FEI TECNAI G2 T-20S Transmission Electron Microscope (FEI company, Hillsboro, OR) at an accelerating voltage of 200 kV.

### Statistical analysis

All statistical analyses were performed using GraphPad Prism version 6.0 (GraphPad Software, La Jolla, CA, USA). The 50% effective concentration (EC_50_) was calculated by non-linear regression analysis. Data sets were analyzed using one-way ANOVA by Dunnett’s test for multiple comparison with a significance of p < 0.05.

## Supplementary information

Supplementary data

## Data Availability

All data generated or analyzed during this study are included in this article (and its Supplementary Information file).
