# Supplementary material for: Neutralization of Acidic Intracellular Vesicles by Niclosamide Inhibits Multiple Steps of the Dengue Virus Life Cycle In Vitro
Source: Sci Rep. 2019 Jun 18;9:8682. doi: 10.1038/s41598-019-45095-1 (PMC6582152; doi:10.1038/s41598-019-45095-1)
Supplement: Supplementary file 1 — Supplementary data [file 41598_2019_45095_MOESM1_ESM.pdf]

Supplementary Information

Neutralization of Acidic Intracellular Vesicles by Niclosamide Inhibits Multiple Steps of the Dengue Virus Life Cycle *In Vitro*

Eunhye Jung[1§], Sangwoo Nam[1,6§], Hyeryeon Oh[1], Sangmi Jun[2,3,7], Hyun-Joo Ro [2,3,7], Baek Kim[4,5], Meehyein Kim[1,6], Yun Young Go[1,6*]

[1]Virus Research Group, Korea Research Institute of Chemical Technology, Daejeon, Republic of Korea

[2]Convergent Research Center for Emerging Virus Infection, Korea Research Institute of Chemical Technology, Daejeon, Republic of Korea

[3]Drug and Disease Target Group, Korea Basic Science Institute, Cheongju, Republic of Korea

[4]Center for Drug Discovery, Department of Pediatrics, School of Medicine, Emory University, Atlanta, GA, 30322, USA

[5]Department of Pharmacy, College of Pharmacy, Kyung Hee University, Seoul, Republic of Korea

[6]Department of Medicinal Chemistry and Pharmacology, University of Science and Technology, Daejeon, Republic of Korea

[7]Bio-Analytical Science, University of Science and Technology, Daejeon, Republic of Korea

*Corresponding author: Yun Young Go; e-mail: yygo@krict.re.kr

[§]Equal contribution

Supplementary Figure 1

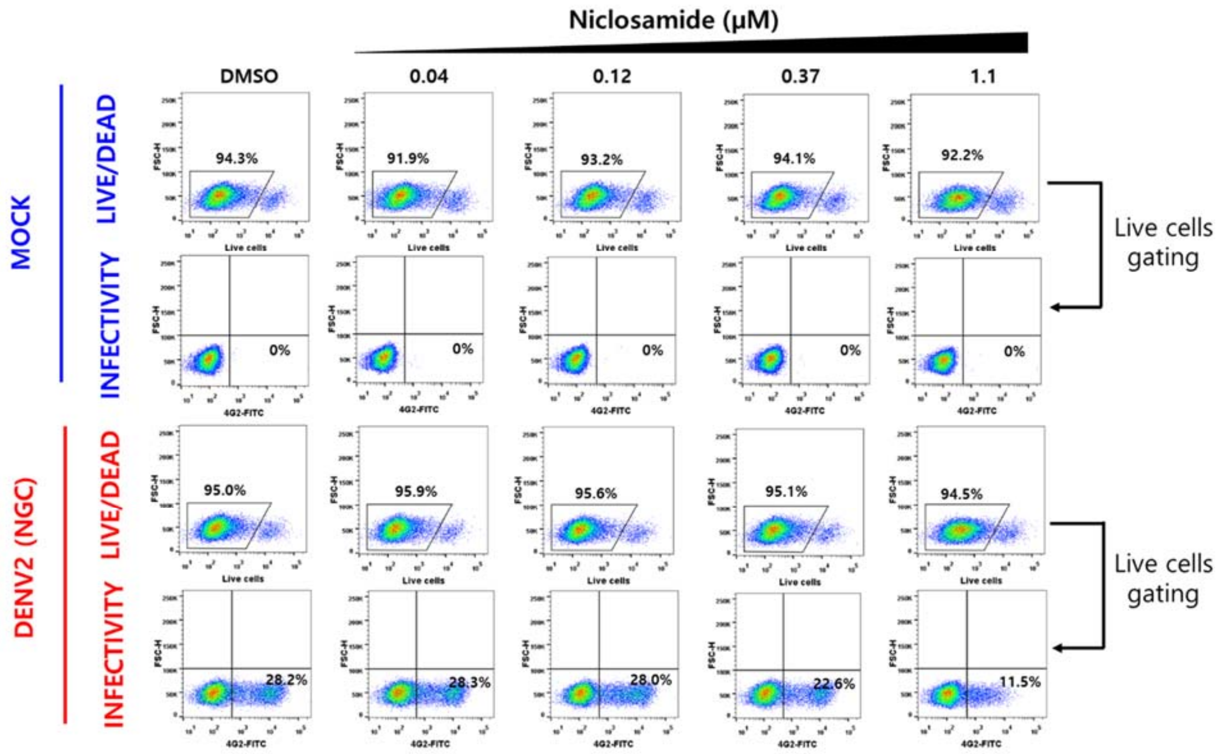

Figure S1. Evaluation of percentage of live/dead cells for mock- and DENV-2 (NGC)-infected Huh-7 cells with increasing concentrations of niclosamide by flow cytometry analysis. Live cells are gated and the percentages are indicated. The DENV-positive cell population is shown in the lower right quadrant.

Supplementary Figure 2

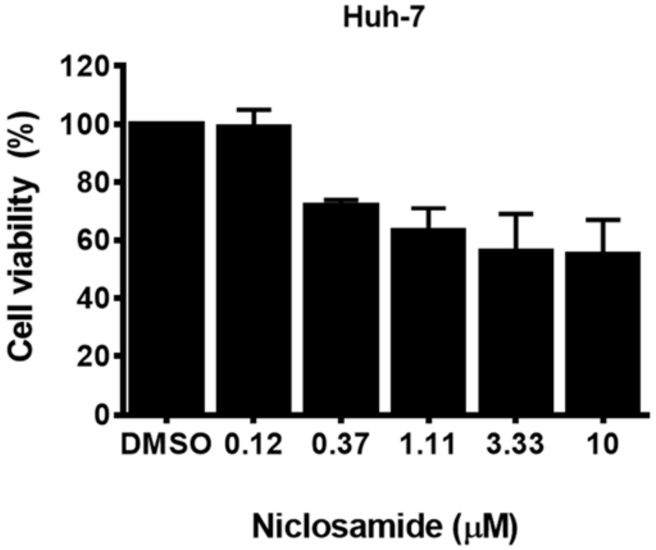

Figure S2. Huh-7 cells were treated with serial-dilutions of niclosamide for 24 h. Cell viability was measured using CellTiter 96 Non-radioactive Cell Proliferation Assay. The data represent means (± SD) of at least two independent experiments performed in duplicate.

Supplementary Figure 3

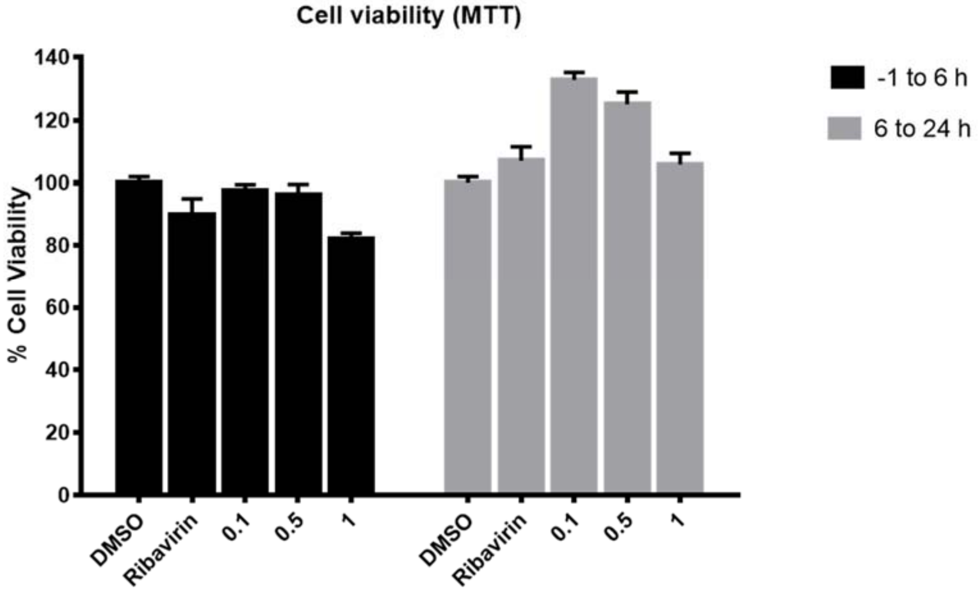

Figure S3. BHK-D2-Fluc replicon cells were treated with different concentrations of niclosamide at indicated times. DMSO was used as solvent control. Cell viability was measured using CellTiter 96 Non-radioactive Cell Proliferation Assay. The data represent means (± SD) of at least two independent experiments performed in duplicate.

Supplementary Figure 4

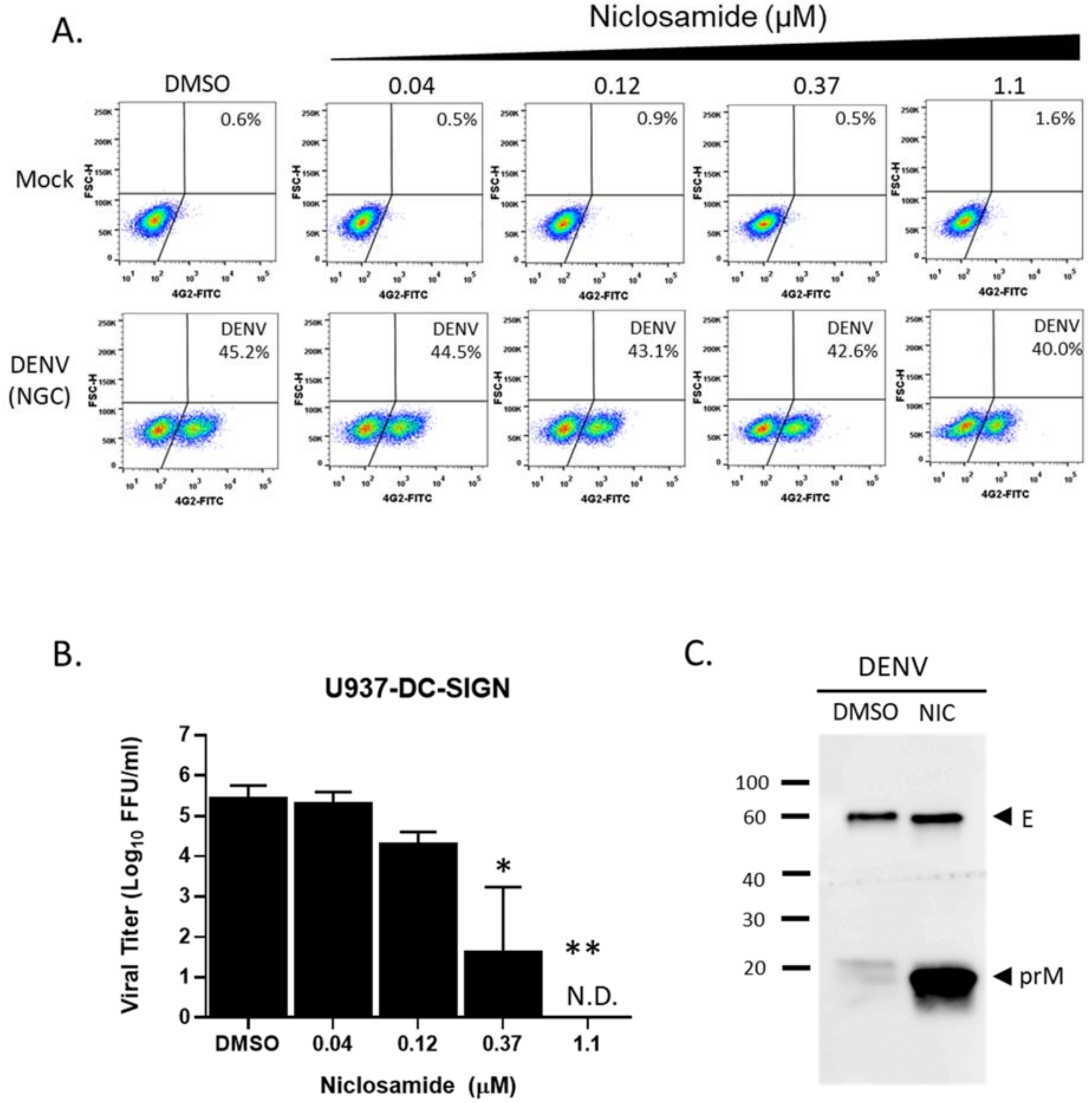

Figure S4. Niclosamide-induced endosomal pH neutralization impairs the maturation of DENV in U937-DC-SIGN cells. (A) Representative dot plot analysis (FSC x 4G2-AF488) of U937-DC-SIGN cells (human lymphocytes expressing dendritic cell-specific intercellular adhesion molecule 3-grabbing non-integrin, ATCC® CRL-3253™) mock-infected (upper panels) or infected with DENV-2 (NGC strain, lower panels) in the presence of the indicated concentrations of niclosamide. The DENV-positive cell population is shown in the lower right quadrant. **(B)** Virus titres from cell culture supernatants were determined with a plaque assay. The data represent the means (± SD) of at least two independent experiments performed in duplicate. N.D., not detected. **(C)** Western blot analysis of prM and E proteins of DENV purified from culture supernatants treated with niclosamide or DMSO. Uncropped image is provided in the supplemental material. * $p < 0.05$ and ** $p < 0.01$ compared to DMSO control.

Uncropped Western blot images

Figure 2E

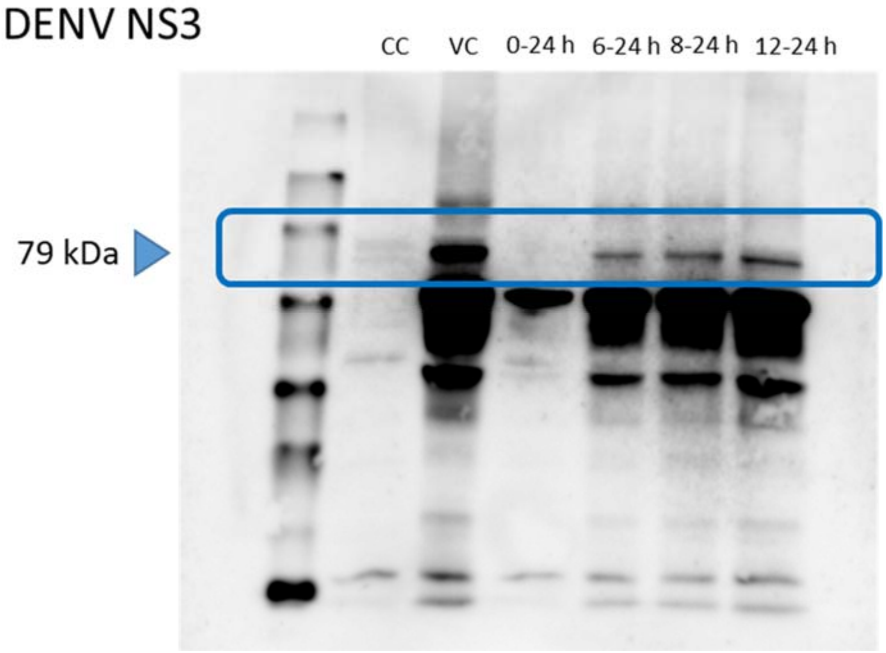

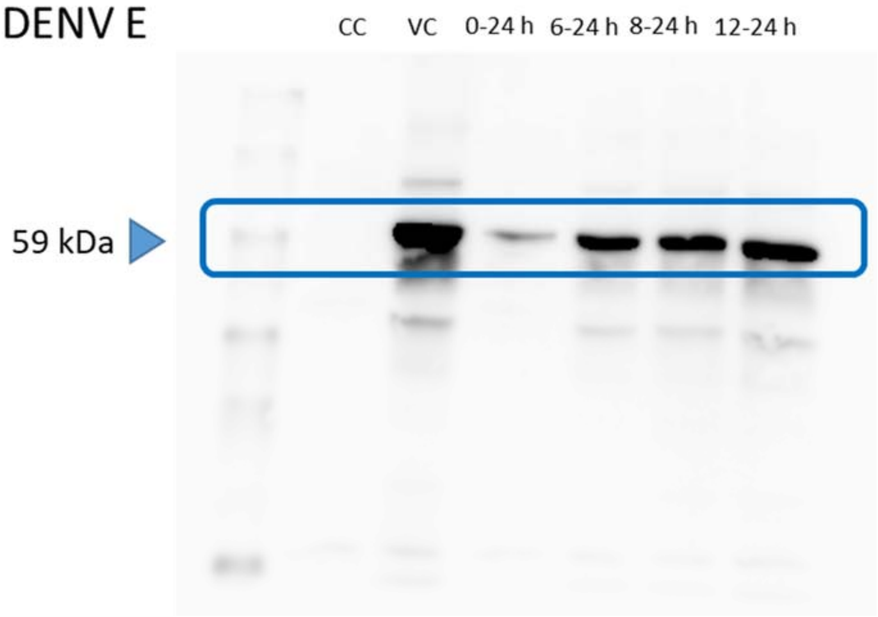

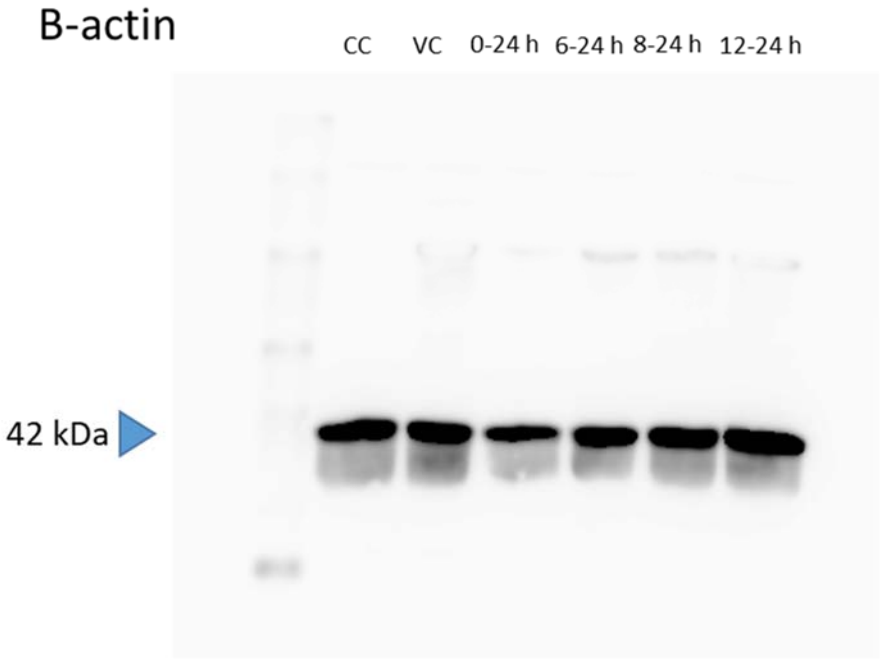
B-actin
CC
VC
0-24 h
6-24 h
8-24 h
12-24 h
42 kDa

Figure 3F

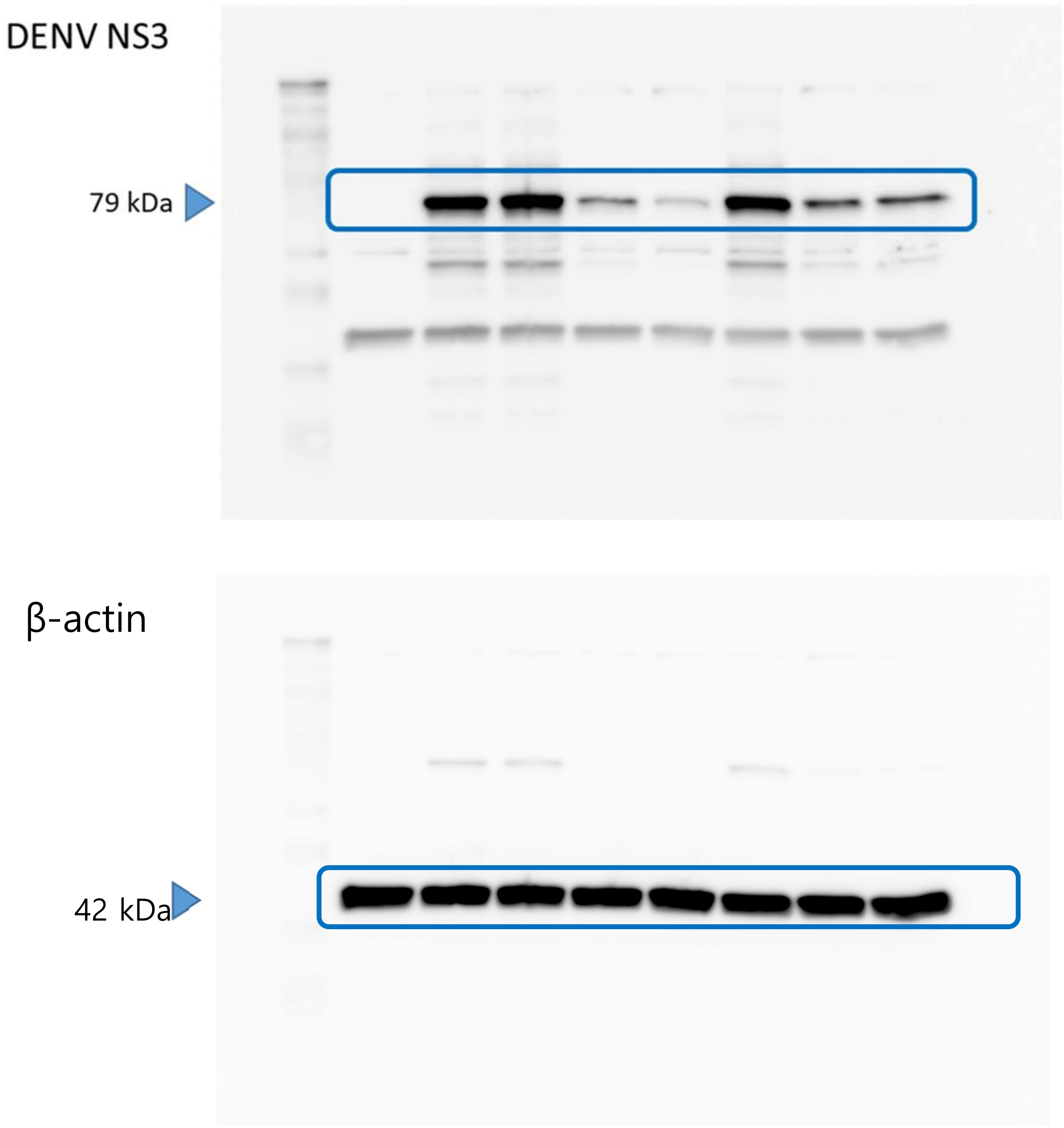

Figure 4A

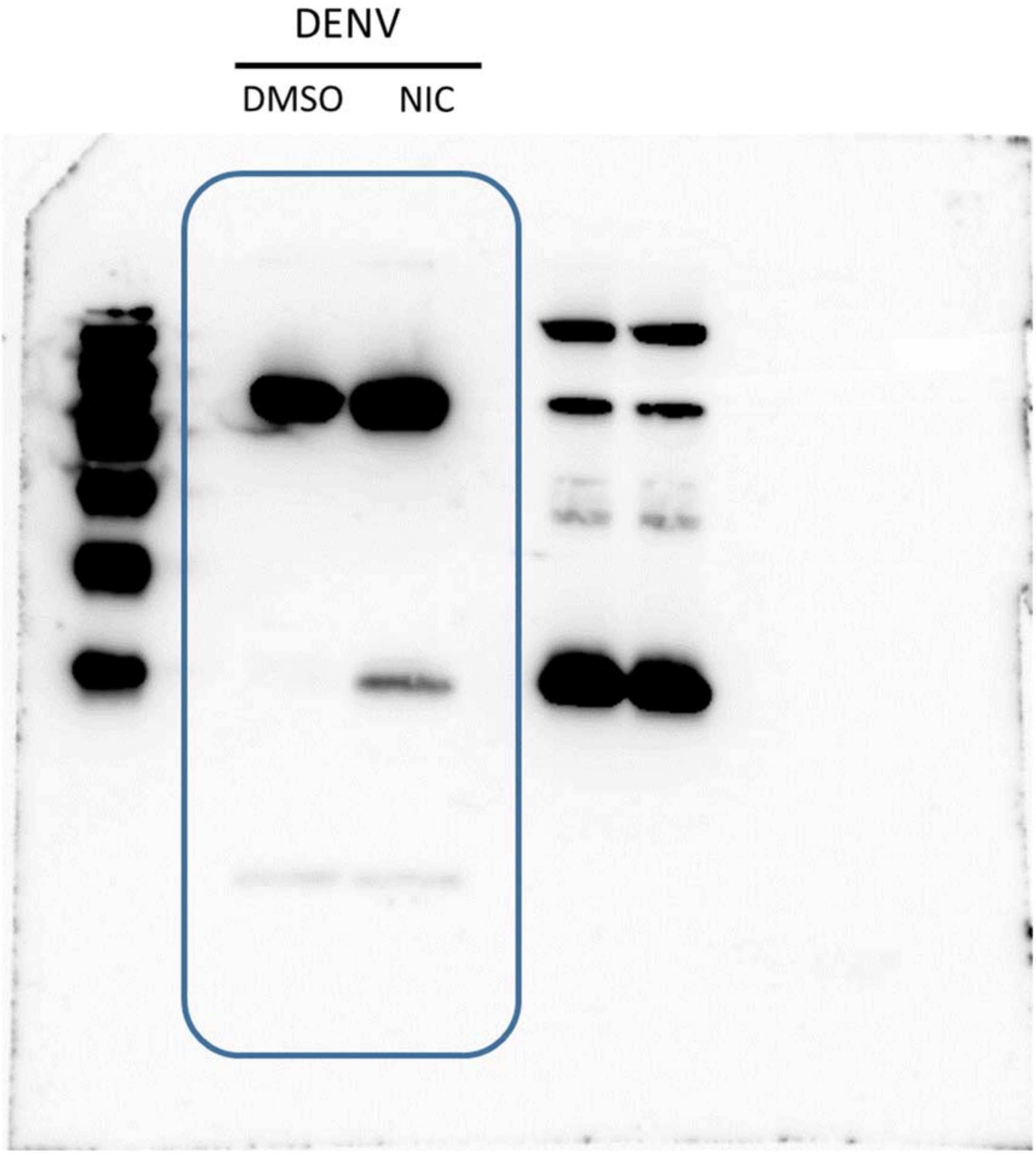

Figure 5C

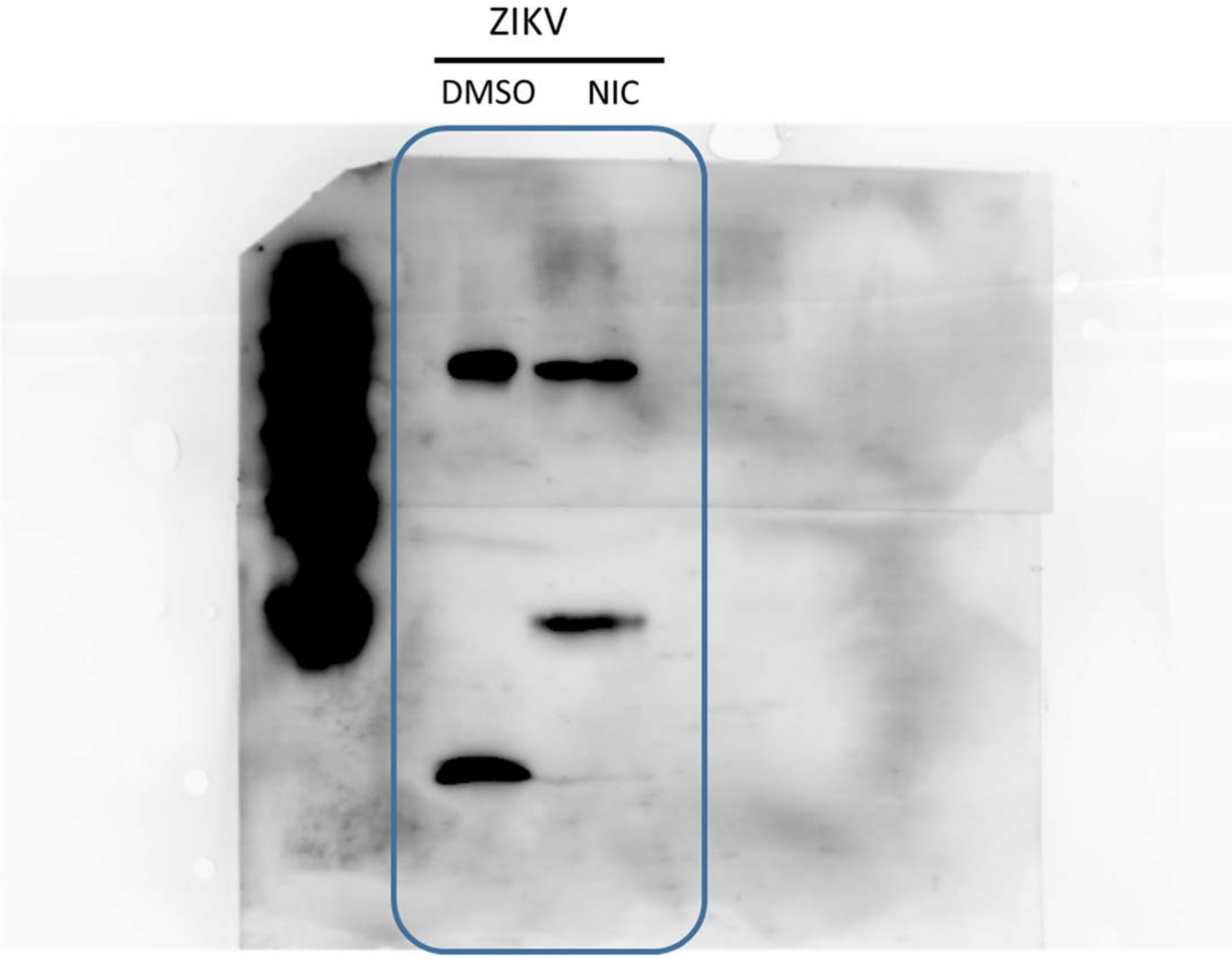

Supplementary Figure S4

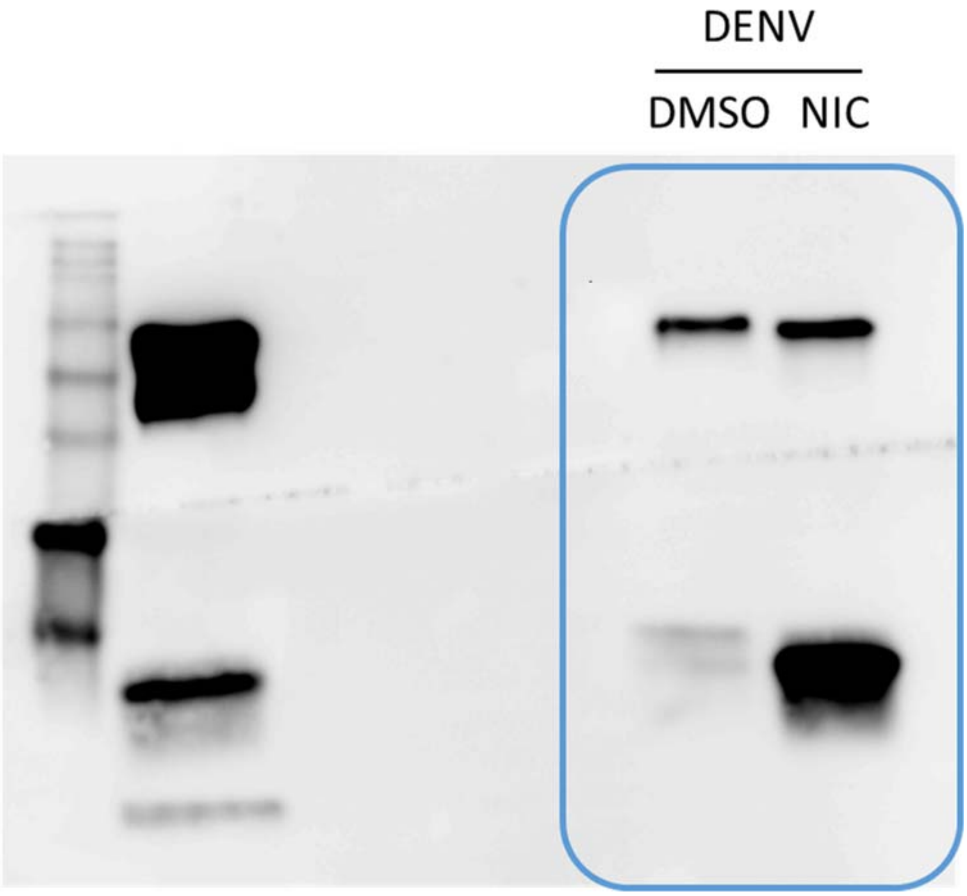

Materials and Methods

Antibodies and reagents. DENV E protein-specific mouse monoclonal antibody (4G2) was prepared from hybridoma cell lines (ATCC® HB-112, ATCC). The anti-dsRNA (Scicons, Szirák, Hungary) monoclonal antibody was used for immunofluorescence analysis. Secondary anti-mouse antibody conjugated to Alexa Fluor 488 (AF488) was obtained from Molecular Probes (Invitrogen). Mouse anti-DENV complex (EMD Millipore, Billerica, MA, USA) and mouse anti-DENV NS1 (Abcam, Cambridge, UK) antibodies were used in focus forming assay for DENV detection. Rabbit anti- DENV prM (GeneTex, Irvine, CA, USA), mouse anti-DENV E (Abcam), and mouse anti-DENV NS3 (Sigma-Aldrich), anti-ZIKV E (GeneTex, Irvine, CA, USA), anti-ZIKV prM (GeneTex) and anti-β-actin (loading control; Sigma-Aldrich) antibodies were used for Western blot analysis. Horseradish peroxidase (HRP)-conjugated goat anti-rabbit and anti-mouse secondary antibodies were purchased from Thermo Scientific (Waltham, MA, USA). Ammonium chloride (NH_4Cl), bafilomycin A1 (BafA1), and niclosamide were purchased from Sigma-Aldrich (St. Louis, MO, USA).

Cell viability assay. Cell viability was determined using CellTiter 96 Non-radioactive Cell Proliferation Assay following manufacturer's instructions (MTT; Promega, WI, USA) The absorbance values were measured at 570 nm using a multi-plate reader (Biotek, Winooski, VT, USA). The 50% cytotoxic concentration (CC_{50}) was defined as the compound's concentration (μM) required for the reduction of cell viability by 50%, which was calculated by regression analysis using Prism v 6.0 (GraphPad Software, La Jolla, CA, USA).
